# Supplementary material for: Bayesian tomography of high-dimensional on-chip biphoton frequency combs with randomized measurements
Source: Nat Commun. 2022 Jul 27;13:4338. doi: 10.1038/s41467-022-31639-z (PMC9329349; doi:10.1038/s41467-022-31639-z)
Supplement: Supplementary file 1 — Peer Review File [file 41467_2022_31639_MOESM1_ESM.pdf]

nature portfolio

Peer Review File

Bayesian tomography of high-dimensional on-chip biphoton frequency combs with randomized measurements

Open Access This file is licensed under a Creative Commons Attribution 4.0 International License, which permits use, sharing, adaptation, distribution and reproduction in any medium or format, as long as you give appropriate credit to the original author(s) and the source, provide a link to the Creative Commons license, and indicate if changes were made. In the cases where the authors are anonymous, such as is the case for the reports of anonymous peer reviewers, author attribution should be to 'Anonymous Referee' followed by a clear attribution to the source work. The images or other third party material in this file are included in the article's Creative Commons license, unless indicated otherwise in a credit line to the material. If material is not included in the article's Creative Commons license and your intended use is not permitted by statutory regulation or exceeds the permitted use, you will need to obtain permission directly from the copyright holder. To view a copy of this license, visit http://creativecommons.org/licenses/by/4.0/.

Editorial Note: This manuscript has been previously reviewed at another journal that is not operating a transparent peer review scheme. This document only contains reviewer comments and rebuttal letters for versions considered at *Nature Communications*.

REVIEWERS' COMMENTS

Reviewer #1 (Remarks to the Author):

I would like to thank the authors for their careful resubmission and response to all my queries. Indeed, my biggest concern was the local nature of the measurement precluding any separated implementation of frequency superposition measurements. As the authors explained, this is not actually the case, as one could synchronise two EOMs to implement spatially separated measurements. In the context of QKD it is then interesting to see how these synchonisations can be done without giving away the measurement basis, but that is certainly a topic for further work and beyond the scope of the paper. I also saw the responses to Reviewer 2 and in my opinion these have been addressed satisfactorially.

As I was already tending to recommend the paper for acceptance in Nature Photonics and the authors have clarified all important issues to me, I am now happy to strongly suggest publication in Nature communications.

Reviewer #2 (Remarks to the Author):

I am happy with the detailed responses provided by the authors to all of my queries and am supportive of publication in Nature Comms.
